# Editorial: Epigenetics and cellular plasticity in glioblastoma

**DOI:** 10.3389/fonc.2023.1179214

**Published:** 2023-03-20

**Authors:** Hui-Wen Lo, Nikos Tapinos

**Affiliations:** ^1^ Department of Neurosurgery, McGovern Medical School, The University of Texas Health Science Center, Houston, TX, United States; ^2^ Department of Neuroscience, Brown University, Providence, RI, United States; ^3^ Laboratory of Cancer Epigenetics and Plasticity, Department of Neuroscience, Brown University, Providence, RI, United States

**Keywords:** epigenetics, cancer stem cell, plasticity, poised, glioblastoma

Glioblastoma (GBM) is the most lethal and prevalent primary brain tumor in adults with a five-year survival rate of only around 5 percent, afflicting about 3.19 people in every 100,000 (1). While chemotherapy, radiotherapy, and surgical removal are offered as current standard treatments, these tumors have a high probability of recurrence, demanding improved treatment options (2). It is well established that GBM’s aggressiveness, treatment resistance, and recurrence is mostly driven by a subset of cells within the tumor bulk that exhibit stem cell properties (3–6). GBM stem cells (GSCs), originate mainly from subventricular zone (SVZ) neural stem cells that have acquired low-level oncogenic driver mutations (7). Although SVZ neural stem cells can undergo fate specific lineage restriction following exposure to differentiation cues (8, 9), GSCs fail to assume complete lineage specification and instead maintain some level of stemness potential (10–12).

Epigenetics and epitranscriptomics—molecular changes of cellular DNA, chromatin, and RNA—can alter the levels of gene transcripts and subsequently change the amount of proteins produced, including those responsible for tumor suppression, progression, or maintenance (13). Until now, there is limited understanding regarding the epigenetic factors that define GSCs’ failure to attenuate their stemness potential in the face of differentiation cues. Origin and maintenance of GSC plasticity are regulated by intrinsic cell processes affecting DNA, chromatin, and RNA of GSCs, as well as by extrinsic factors of the tumor microenvironment that propagate cancer stem cell phenotypes. Both intrinsic and extrinsic epigenetic pressure contribute to the ability of GSCs to remain plastic, maintain a poised state, and constantly interconvert between more and less differentiated states which, allows GSCs and differentiated progenies to adopt a population equilibrium that facilitates tumor persistence (10, 12, 14).

In the early 2000’s, Helen Blau et.al., introduced an evolving concept for adult stem cells suggesting that stem cells is rather a biological function instead of an entity (15). According to this view, at least some stem cells in adult tissues are highly plastic and amenable to changes when given the appropriate microenvironment. GBM is a perfect example of cancer evolution where cells retain an inherent level of plasticity through activation or maintenance of progenitor developmental programs. Thus, the concept of GSC as a function of GBM cancer cells rather than a separate entity could help explain the heterogeneity and cellular adaptation of these tumors. In such model, GSCs are interconverting between stem cell and poised stem cell stages through the constant influence of intrinsic and extrinsic epigenetic mechanisms (**Figure 1**). Studies of the GSC epigenome, epitranscriptome, non-coding RNome and 3D genome architecture can reshape our view of GBM tumor evolution and inform the design of new epigenetic targeting therapies.

**Figure 1:**
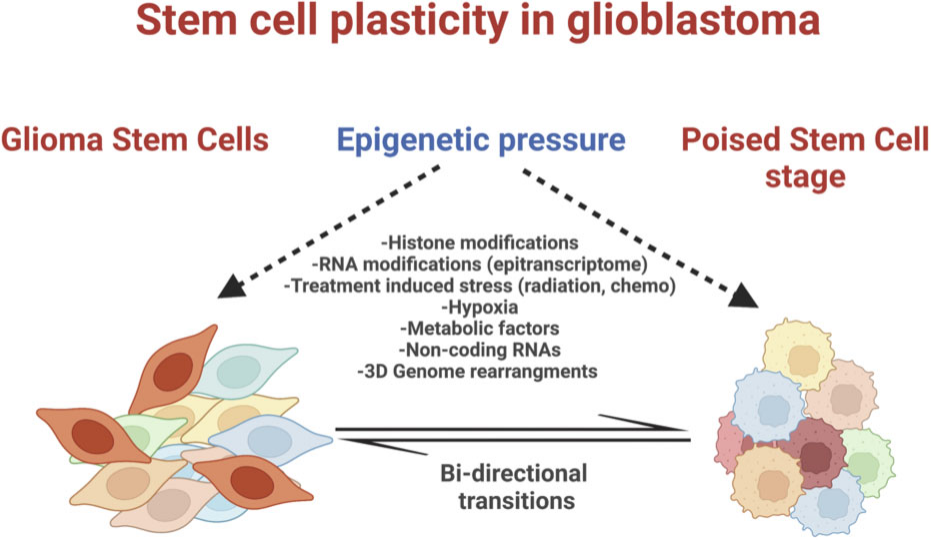
Intrinsic and extrinsic epigenetic pressure maintains GBM cells at an adaptive or "poised" stem cell stage.

This Research Topic includes 5 original research papers. Yu et al., combined analysis of transcriptome and DNA methylation profiles of TCGA to derive a 6-gene model-based risk score. This approach allowed the authors to predict survival and the role of personalized treatment plans for GBM. Wang et al., analyzed tumor associated fibroblasts infiltration by Estimating the Proportion of Immune and Cancer cells (EPIC) based on multiple glioma databases. They describe a novel transcript signature that predicts glioma prognosis. Shi et al., showed that individual glioblastoma cell lines displayed increased expression of the short splice variant of YKL-40 after low serum treatment. In addition, unlike the full-length (FL) version, which was localized to all cell compartments, the short isoform could not be secreted and was localized only to the cytoplasm. Functionally, FL YKL-40 promoted cell proliferation and migration, whereas SV YKL-40 suppressed them. The authors described pathways that regulate the expression of the SV YKL-40 and discuss the significance for development of new therapies. Majc et al., investigated the use of bioactive peptides from venoms as novel therapies targeting cancer specific pathways in glioblastoma. Finally, Basilico et al., revealed that changing the rigidity of the mechanical environment tuned the response of glioblastoma cell lines through change in morphological features, epithelial-mesenchymal markers (E-, N-Cadherin), EGFR and ROS expressions in an interrelated manner. Their work highlights the importance of microenvironment stiffness in the regulation of glioblastoma invasive properties.

## Author contributions

NT wrote the initial draft of the editorial. H-WL edited and both agreed on the final version.
